# Clinical Experience of Fluroscopy Guided Percutaneous Transpedicular Biopsy of Spinal Lesion

**DOI:** 10.31729/jnma.3950

**Published:** 2019-02-28

**Authors:** Suman Rijal, Pankaj Raj Nepal, Manita Raut, Dinesh Nath Gongal

**Affiliations:** 1 Department of Neurosurgery, Upendra Devkota Memorial National Institute of Neurological and Allied Sciences, Bansbari, Kathmandu, Nepal; 2 Department of Neurosurgery, B and C Medical College and Teaching Hospital, Birtamod, Jhapa, Nepal

**Keywords:** *fluoroscopy guided*, *metastasis*, *spinal lesions*, *transpedicular biopsy*, *Tuberculosis*

## Abstract

**Introduction:**

The diagnosis of the spinal lesions often puts the clinician in dilemma. The definite diagnosis is obviously needed for the proper management of the disease. The wrong diagnosis not only imposes the adverse effects, but sometimes may lead to the disabling conditions and even prove to be life threatening. This study is aimed at evaluating the need of fluoroscopy guided percutaneous transpedicular biopsy for establishing the proper diagnosis and find the diagnostic yield.

**Methods:**

This is the descriptive cross-sectional study conducted over the period of 10 years in the Upendra Devkota Memorial National Institute of Neurological and Allied Sciences among the patients who underwent transpedicular biopsy for various spinal lesions.

**Results:**

Among the 77 cases, 38 (49%) of the lesions on MRI were single level whereas 39 (51%) of the lesions were multiple. Most of the lesions were diagnosed as the non-tubercular infection 30 (42%), followed by the osteoporotic fractures and malignancy in 18 (25%) and 15 (21%) respectively. The sensitivity and specificity of the radiology with the background of clinical scenario was 79.5% and 90.9% respectively. The diagnostic yield of the biopsy was 93.5%.

**Conclusions:**

The transpedicular biopsy of the spinal lesion is the must for the proper diagnosis and treatment plan of such cases. The change in the diagnosis after biopsy is often possible which will drastically alter the treatment plan.

## INTRODUCTION

Diagnosis of the various spinal lesions has been increasing due to the upfront imaging technology and the reach of patients to the medical care. Uncertainty in diagnosis may lead to the wrong treatment in those patients.^1^ Definitive histopathological diagnosis of spinal lesion is eminent to standardized management strategy.

There has been extensive study regarding the efficacy of the transpedicular vertebral body biopsy (TPVB) of the spinal lesions in the past.^2,3^ However, there are sparse literature in the setting of developing country like Nepal.

With the aim to establish the efficacy of biopsy of the various spinal lesions by means of TPVB, this study has been conducted in the single tertiary neurosurgical care center.

## METHODS

This is a descriptive cross-sectional study conducted in Upendra Devkota Memorial National Institute of Neurological and Allied Sciences, Bansbari, Kathmandu, Nepal among 77 patients who underwent TPVB of spinal lesion during a period of 10 years from 2008-2017. Ethical approval was taken from UDMNINAS, IRC. Patient who underwent open biopsy or decompression of cord were excluded. Age, gender, site of lesion, clinical findings, etiology, tuberculin test, radiological diagnosis, histopathological examination (HPE) reports, complication and mortality data were retrieved from medical archives. Sensitivity and specificity of radiological finding and diagnostic yield of TPVB was analyzed.

## RESULTS

There were 77 patients included in the study. Among them 32 (42%) were male and 45 (58%) were female. The mean age was 54.43 (SD 16.89) years. Of all the cases, in 38 (49%) patients the lesion was single and in 39 (51%) cases the lesions were multiple. Of the multiple lesions, 32 (82%) lesions were contiguous and 7 (18%) were skip lesions (Table 1).

**Table 1.** Lesion distribution according to the multiplicity.

S.N	Multiplicity of Lesion	n (%)
1.	Single	38 (49%)
	Multiple	39 (51%)
2		
	a. Contiguous lesion	32 (82%)
	b. Skip lesions	7 (18%)

**Figure 1.**
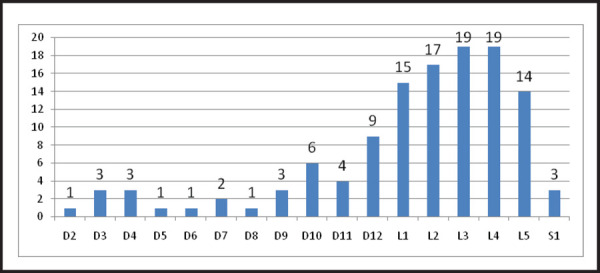
Topographic distribution of vertebral body lesion.

Moreover, most of the lesions that involved the vertebral body was confined to lumbar vertebra 84 (69.4%) with few distributed to the thoracic region 34 (28.1%) and sacral region 3 (2.5%) (Figure 1).

**Figure 2.**
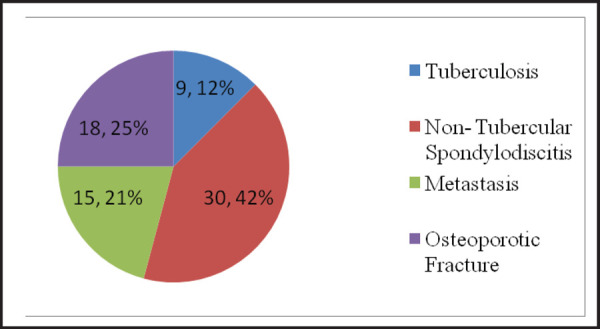
Histopathological diagnosis of spinal lesions.

Among the 19 cases with disc space involvement, 17 cases had involvement of the intervertebral disc space at the lumbar region. Of all the vertebral biopsy undertaken, 32 (42%) were proved to be non-tubercular spondylodiscitis followed by osteoporotic fracture 18 (25%) and metastatic lesion 15 (21%) respectively. Moreover, tuberculosis was revealed in 9 (12%) of the biopsy (Figure 2).

Only 7 out of 26 with tuberculin test positive were proved to be tuberculosis by HPE. Also, 2 cases out of 51 with negative tuberculin test came to be tuberculosis in HPE. The sensitivity and specificity of tuberculin test in diagnosing spinal tuberculosis was 77.80% and 72.10% respectively.

Radiological diagnosis of vertebral infection was refuted by TPVB among 20.5%. Whereas, radiological diagnosis of vertebral infection was verified by TPVB in 79.5% of the cases. The sensitivity and specificity of the radiology in diagnosing infection were 79.50% and 90.90% respectively (Table 2).

**Table 2.** Accuracy of radiology in diagnosing infection.

		HPE Prover Infection		
				Total
		Positive	Negative	
Radiological	Negative	30	8	38
Infection	Positive	3	31	34
Total		33	39	72
Sensitivity: 79.50%, Specificity: 90.90%				

Out of 17 radiological suspicion of metastasis (Mets), 11 were ascertained histopathologically. Similarly, out of 27 radiological suspicion of pyogenic infection, 17 were ascertained histopathologically to be same. However, only 2 cases were proved to be tubercular, out of the 8 radiological suspicions. Moreover, 12 out of 17 cases suspected to have osteoporotic fracture were proved by TPVB. Radiology was inconclusive to report specific diagnosis in 8 cases. Among those undetermined cases, 5 cases were proved to be of pyogenic infection, 2 were proved to be metastasis.

However, HPE was reported normal in one case.

Of the 77 total cases, 5 cases had normal finding in HPE (Table 3). The diagnostic yield of TPVB was 93.5%.

**Table 3.** Comparison of radiological diagnosis with HPE report.

	Histopathology reports						
		Normal	Pyogenic	TB	Mets	Osteoporotic	Total
	Mets	2	1	0	11	3	17
Initial radiological diagnosis	Pyogenic	1	17	6	0	3	27
	TB	0	6	2	0	0	8
	Osteoporotic	1	1	1	2	12	17
	Undetermined	1	5	0	2	0	8
	Total	5	30	9	15	18	77
*Diagnostic yield of TPVB= 93.5%.*							
*Preoperative radiological diagnosis was static among 55.85%.*							
*Change in preoperative diagnosis by TPVB among 44.15%.*							

Among all the cases, we experienced hemothorax as the complication of TPVB at thoracic level in one patient, which was managed with chest tube insertion. No other complications were noted. There was no mortality in our series.

## DISCUSSION

There has been extensive study regarding the efficacy of the TPVB of the spinal lesions in the past.^2,3^ As of our study, the uncertainty of the diagnosis only by means of clinical and radiological findings has been found in the study done by Gupta et al.^1^ Such kind of uncertain diagnosis may lead to the wrong treatment in those patients. The study done by Smith et al., showed that MRI findings of the tubercular origin are similar to that of neoplasms.^4^ Furthermore, the need of establishing the diagnosis by biopsy has also been emphasized by many other authers.^1,5^ The various limitations that differentiate the malignant pathological fracture and the osteoporotic fractures has been highlighted by Pozzi et al.^6^

Our study demonstrated all tubercular looking like spinal lesions in the MR are not always tubercular. Rather, few lesions came out to be metastatic and osteoporotic fractures along with few being the infectious too and vice-versa. The study done by Janardhana also showed that 17% of the cases had change in diagnosis after the biopsy.^7^ We found that the tuberculin test is not very specific and sensitive in diagnosing the tubercular lesion. This fact clearly demonstrates the need of biopsy in any spinal lesions. The biopsy has got high sensitivity in diagnosing the malignancy and the infection. Rivas et al. had found the sensitivity and specificity of the biopsy as 86% and 100% respectively.^8^ In our study, the sensitivity and specificity of the radiology was 79.5% and 90.90% respectively. Moreover, the diagnostic yield of the transpedicular biopsy was 93.5%.

Various complications of the transpedicular biopsy have been reported in the literature and have been estimated from 0 to 7.6%.^2,9^ The older paper had reported the complication rate up to 26%.^10^ In our study only one patient who had thoracic vertebra transpedicular biopsy had hemothorax which was successfully managed with the chest tube insertion. No other complications were noted in our study. CT or the fluoroscopy has made this invasive procedure more safer and accurate.^11,12^

## CONCLUSIONS

The fluoroscopy guided TPVB is safe and efficacious tool to target the spinal lesion with high accuracy. Prospective study including larger population is recommended to support this conclusion.

## Conflict of Interest

**None.**
